# Human Papillomavirus and Cancers

**DOI:** 10.3390/cancers12123772

**Published:** 2020-12-15

**Authors:** Maria Lina Tornesello, Franco M. Buonaguro

**Affiliations:** Molecular Biology and Viral Oncology Unit, Istituto Nazionale Tumori IRCCS “Fondazione G. Pascale”, via Mariano Semmola, 80131 Naples, Italy

Persistent infection with oncogenic human papillomaviruses (HPVs) is the main cause of nearly all cervical cancers as well as of a significant proportion of other malignancies arising from the mucosal squamous epithelia of the anogenital tract as well as of the head and neck region [1]. While HPV vaccination programs will have a great impact on the global prevention of cervical neoplasia, other HPV-related cancers will continue to be a serious health problem for some decades to come [2]. Many efforts are still necessary to understand the complex interplay between viral and host factors and to find the best approach for prevention, early diagnosis, and treatment of HPV-related diseases.

In the Special Issue titled “Human papillomavirus (HPV) and cancer”, several experts from all over the world have reported and discussed the most recent advances from basic science to clinical management of HPV-related malignancies. A special focus has been given to the oncogenic role of epigenetic factors, viral proteins and immune response in HPV-driven cancers, as well as to the new anti-cancer opportunities including HPV-based therapeutic vaccines.

Novel classes of non-coding RNAs (ncRNAs), such as circular RNAs (circRNAs), pico RNAs (piRNAs) and long noncoding RNAs (lncRNAs), have been found deregulated differently in diverse histotypes of HPV-driven tumours. Casarotto et al. in their review reported that two oncogenic circRNAs are over-expressed and able to sponge specific miRNAs in HPV-positive cervical cancer and derived cell lines [3]. On the other hand, a large number of piRNAs have been found differently expressed in HPV-positive and HPV-negative head and neck squamous cell carcinoma (HNSCC). Among these, the expression of five piRNAs has shown to be associated with worse overall survival in viral-driven HNSCC. PiRNAs, similar only in size to miRNAs, are able to associate specifically with P-element-induced wimpy testis (PIWI) proteins causing epigenetic changes that are important for cell transformation [3].

The role of HPV infection in HNSCC and its significance as a prognostic marker that is indicative of clinical outcome has emerged in the last few decades [4]. The incidence of HPV-driven HNSCC varies in diverse geographical regions [5]. Alsbeih et al. investigated the HPV distribution and its prognostic value in HNSCC of Saudi patients [6]. The results of the study showed that HPV prevalence is significantly lower in Saudi HNSCC than in other parts of the world and, consistent with studies performed in other countries, they observed that patients with HPV/p16 positive tumours had a better overall survival.

The alteration of metabolism in cancer cells is crucial for tumour growth; however, the effect of HPV infection on metabolic pathways has not been well characterized in HNSCC. Prusinkiewicz et al. analysed the RNAseq profiles of HPV-positive and HPV-negative HNSCCs retrieved from the TCGA and identified many differentially expressed metabolic genes between the two cancer groups [7]. Importantly, genes involved in glycolysis were down-regulated while those involved in the tricarboxylic acid cycle, oxidative phosphorylation, and β-oxidation were up-regulated in HPV-positive HNSCC compared to HPV-negative HNSCC. The reduced expression of several genes was predictive of better survival in patients with HPV-positive HNSCC.

Many biomarkers have been identified in HPV-negative HNSCC; however, few of them have been validated for clinical use. Mittal et al. investigated the prognostic role of centrosome amplification in HPV-negative oropharyngeal SCC (OPSCC) [8]. Centrosome amplification was found more expressed in HPV-negative compared to HPV-positive OPSCC biopsies and associated with poor overall survival. Therefore, centrosome amplification may serve as a novel prognostic biomarker for patients with HPV-negative OPSCC.

The expression of cell cycle regulators such as D-type cyclins has been found frequently deregulated in HNSCC. Novotný et al. performed a comparative analysis of paired tumour/peri-tumour tissues and showed that cyclin D1 was upregulated in 18% of HNSCC and downregulated in 23% of carcinomas, mainly in HPV-positive samples [9]. Moreover, the change in CCND1 expression was observed to be compensated by cyclin D2 expression independently from the HPV status.

The class II major histocompatibility complex (MHC class II) molecules become expressed on the surface of epithelial cells during the inflammation process and they can function as antigen presenting cells (APCs). Gameiro et al. analysed the HNSCC RNA-seq data retrieved from TCGA in order to determine the effect of HPV on the expression of MHC-II genes and other immune related genes [10]. All MHC-II genes along with genes encoding various co-stimulatory molecules involved in T-cell activation were found to be significantly upregulated in HPV-positive tumours compared to HPV-negative HNSCC. Therefore, the highly immunogenic tumour microenvironment observed in HPV-positive HNSCC may be due to the antigen presentation of epithelial cells.

The favourable prognosis of HPV-positive/p16-positive cancer patients, as reported in several clinical trials, demands for de-escalation therapies. However, it is very important to implement HPV testing methods that accurately distinguish HPV-driven OPSCC from HPV-negative tumours. Borena et al. used a sandwich ELISA test to detect the expression of HPV 16, 18 and 45 E7 oncoproteins and compared their results with HPV DNA positivity and p16 immunohistochemistry (IHC) as the reference method [11]. The authors found a significant concordance between E7 oncoprotein detection and the reference method and propose larger studies to confirm the diagnostic value of the assay.

The juvenile-onset recurrent respiratory papillomatosis (JoRRP), caused by the infection with HPV 6 and 11, is a rare and severe respiratory disease that follows an unpredictable clinical course. Lépine et al. analysed viral biomarkers of JoRRP severity by using a chromogenic in situ hybridization (CISH) method detecting the E6 and E7 mRNA of HPV 6 and 11 [12]. They stratified samples in low staining versus high staining and concluded that HPV E6 and E7 CISH might be a biomarker predictive of disease aggressiveness in JoRRP.

Several infectious agents, in addition to oncogenic viruses, may contribute to increase the risk of cancer development. Kofler et al. investigated the role of some sexually transmitted pathogens, including Ureaplasma spp., Chlamydia trachomatis, Mycoplasma hominis, Mycoplasma genitalium and HPV, in oropharyngeal carcinoma [13]. HPV DNA was detected in almost 70% of the oropharyngeal cell exfoliates collected by brushing from OPSCC patients. Conversely, the low prevalence of Ureaplasma spp. and the absence of the other pathogens among patients with oropharyngeal cancer do not support their oncogenic role. Moreover, HPV detection in cell brushing of OPSCC patients following surgery has a prognostic significance. Indeed, seven out of 62 HPV positive patients remained positive at post-treatment follow-up and five had a tumour relapse/progression. Importantly, all HPV-negative patients remained free of disease suggesting the relevance of HPV testing after treatment [14].

Clinical behaviour of HNSCC mainly depends on the site and the HPV status. Numerous studies have shown that virus-related HNSCCs possess peculiar clinical and biological features. Perri et al. described the molecular differences and similarities between HPV-negative and HPV-positive HNSCC including the better prognosis and response to therapies of the latter group of patients [15]. Remarkably, patients with HPV-driven HNSCCs are frequently more responsive to conservative treatments and immunotherapy, opening questions about the use of pre-therapy assessment in order to guide the treatment strategy.

The antiviral agent cidofovir has been previously shown to have an anti-proliferative effect on cervical cancer derived cell lines. Verhees et al. investigated the effect of cidofovir on the growth of HPV-positive and -negative HNSCC-derived cell lines [16]. Cidofovir was able to inhibit the cell proliferation and to cause γ-H2AX accumulation as well as upregulation of DNA repair proteins. The effect was marked in HPV-positive cells and indicative of the occurrence of mitotic catastrophe.

The incidence of HPV-related cancers is significantly higher in HIV-positive subjects compared to the general population and highly active anti-retroviral therapy (HAART) does not seem to have changed the trend. Alam et al. reported that protease inhibitors (PI) caused destabilization of cell-cell junctions within the stratified epithelium of three-dimensional tissues derived from primary human gingiva and cervical epithelial cells [17]. This caused enhanced infectivity of HPV16 to the basal layers and de novo virus biosynthesis suggesting an increased the risk of HPV-driven oral and cervical cancer development in HIV-positive patients under HAART treatment.

Therapeutic targeting of viral oncogenes represents a promising strategy to treat HPV-related cancers. Ehrke-Schulz et al. employed the adenoviral vectors HCAdVs expressing the CRISPR/Cas9 machinery to specifically inactivate the HPV18 or HPV16 E6 genes in HeLa, SiHa and CaSki cervical cancer derived cell lines [18]. Transduced cervical cancer cells showed increased apoptosis and decreased proliferation suggesting that HCAdV can serve as a therapeutic agent when armed with HPV-type-specific CRISPR/Cas9.

Specific antibodies in a single-chain format (scFvs) able to target the E6 and E7 oncoproteins of high-risk HPVs represent new therapeutic molecules against HPV-associated neoplastic lesions. Amici et al. evaluated the antigen-binding capacity of diverse anti-16E7 scFvs by using E7 mutants as well as by performing computational analyses to define length, total net charge, hydrophobicity, polarity, and charge distribution to better adapt the antibodies for clinical use [19]. Hence, scFvs may represent valid candidates for HPV-related cancer immunotherapy.

DNA-based cancer vaccines have a promising application in the prevention as well as in the treatment of diverse types of tumours, including HPV-related cancers. Franconi et al. discussed how the immunogenicity of DNA vaccines can be improved by fusing of HPV antigens with plant gene sequences as well as by using plant-derived immunomodulatory agents [20]. Such molecules have shown to interfere with the processes of carcinogenesis by modulating many different cellular pathways and reducing the drug resistance of tumours.

HPV is the most common sexually transmitted infection in the world and in the large majority of cases, the virus is cleared by the immune system. However, in several cases, HPV is able to escape the immune control and to cause cancer development. Ferreira et al. described several strategies of immune evasion adopted by HPVs and in particular the ability of E6 and E7 to synergistically target almost all cellular innate immune pathways [21].

More than 90% of anal cancers are associated with HPV infection. The study by Carter et al. aimed at identifying cancer-associated circulating cells in peripheral blood of anal cancer patients in order to monitor treatment efficacy and to diagnose relapse [22]. Nucleated cells were isolated from the blood of anal cancer patients and cancer cells identified by using pan-cytokeratin and CD45 antibodies. The successful identification of cancer cells opened new opportunities for the diagnosis and prognosis of anal cancer patients.

The growth of human keratinocytes in three-dimensional (3D) cultures emulating the original stratified epithelium represents an important technical advancement for in vitro studies of HPV-related carcinogenesis. De Gregorio et al., in their review, critically described the diverse in vitro models of HPV-related cancers from the simplified “raft culture” to the complex three-dimensional (3D) organotypic models with a special focus on the artificial tissues containing the tumour microenvironment (TME) components [23]. Novelties in the field of 3D cultures of HPV-associated anogenital and oropharyngeal malignancies have been extensively discussed. Moreover, Maru et al. reported interesting results on the propagation of organoids from the normal squamocolumnar junction (SCJ) cells of the uterine cervix by using Matrigel-based three-dimensional cultures [24]. Such organoids characterized by a dense structure contained mainly squamous cells and in some cases a few mucin-producing endocervix cells. Therefore, such organoids are likely to provide a novel platform to study HPV carcinogenesis in ecto and endocervical cells.

Cervical intraepithelial neoplasia (CIN) may either regress or progress to cervical cancer depending on several factors related to the virus and host interplay. There are no validated biomarkers able to distinguish progressing from regressing neoplastic lesions. Taguchi et al. used a continuous-time multistate Markov model to estimate the bidirectional transition of cervical lesions for designated HPV genotypes [25]. Their model was applied to a retrospective cohort and was able to highlight major differences in the progression patterns between high-risk HPV-related CINs.

Cervical cancer prevention is mainly based on the screening of cytological smears and the treatment of precancerous lesions. The HPV testing in self-sampled cervical exfoliates has been shown to increase the participation of women in oncological screenings particularly in never- or under-screened populations. Hawkes et al., in their review, discussed several studies that have examined the self-collection of cervical samples for HPV-based cervical screening including the collection devices and assays [26]. The authors concluded that self-collection will be crucial for global screening programs and the elimination of cervical cancer as a public health problem.

In conclusion, the HPV and cancer Special Issue covers a substantial portion of the recent knowledge and latest accomplishments in the HPV-related cancer field.
